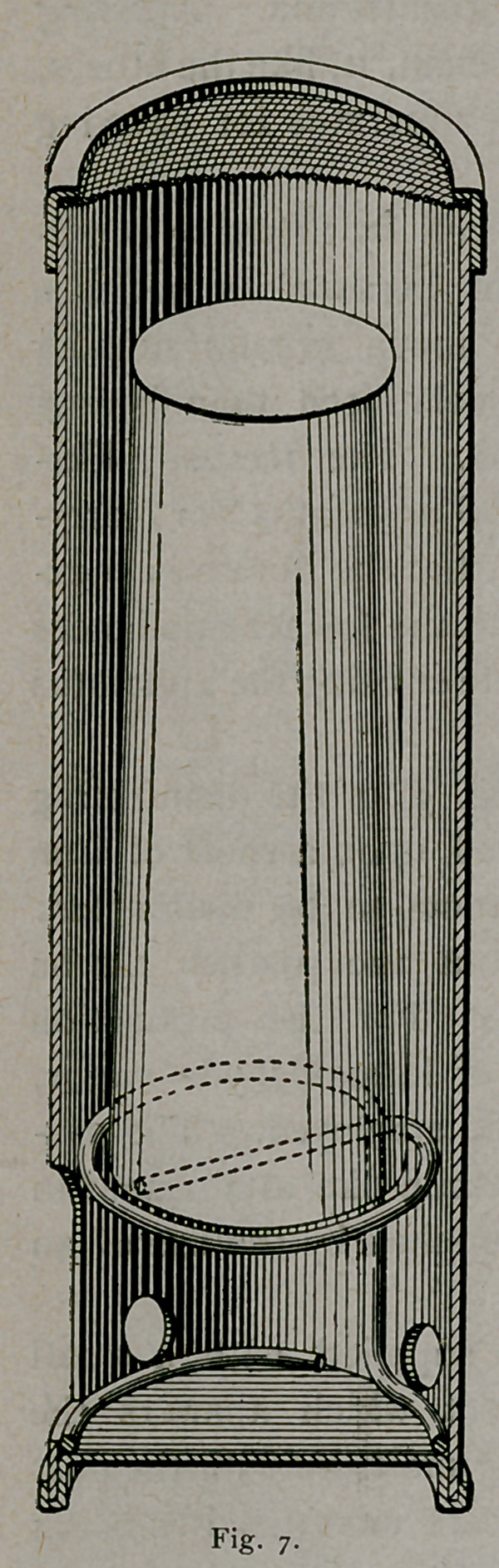# Formaldehyde Gas1Read before the section on pathology, Buffalo Academy of Medicine, January 15, 1901.

**Published:** 1901-02

**Authors:** William G. Bissell

**Affiliations:** Bacteriologist Department ot Health, Buffalo, N.Y.


### FORMALDEHYDE GAS.[1]

### ITS MOST SIMPLE APPLICATION AND ITS LIMITATIONS IN HOUSEHOLD DISINFECTION.

By WILLIAM G. BISSELL, M. D.,
Bacteriologist Department ot Health, Buffalo, N. Y.

AN APOLOGY might seem necessary in offering for discussion a subject which has received such wide attention by sanitary experts, and particularly when I am unable to present anything new regarding the chemistry of formaldehyde as a disinfectant.

The excuse for presenting this subject is the experience gained in the actual application of the various methods for the use of formaldehyde gas in household disinfection, and an attempt to correct many misleading expressions which have been published from time to time in the medical literature and which have influenced the physicians of Buffalo, and probably those of many other cities, also, expect too great results from the application of gaseous substances in the disinfection of the household after the existence of a contagious malady.

1. Read before the section on pathology, Buffalo Academy of Medicine, January 15, 1901.

The thorough disinfection of rooms and their contents constitutes one of the chief means for the prevention of the spread of disease. Up to the time of the introduction of formaldehyde gas, sulphur fumigation was most extensively employed. From time to time, medical literature has contained articles which have been the means of casting doubts upon the efficiency of sulphur dioxide as a germicide, this being due perhaps to excessive expectations of its germicidal value and especially by its improper employment.

The etiological factors in the production of such diseases as measles, smallpox and scarlet fever are unknown. Sulphur dioxide will not kill the spores of anthrax, nor will it so affect certain other resistant germs as to prevent their growth in culture. This fact does not disprove that sulphur dioxide accomplishes much in rendering noninfective apartments where measles, and the like, have existed. Practical observations demonstrate this point.

When the sulphur method is properly employed, there is no doubt of its efficiency in restricting the spread of many infectious maladies. It is contended by one[1] who is in a position to know that in the practical disinfection of the household it is not always necessary to completely kill the organisms (although this result, if it can be attained, is the best,) but to so decrease their disease-producing powers that they are incapable of producing immediate infection and "factor time," in the majority of instances, will cause complete destruction. The efficiency of sulphur dioxide as a germicidal agent can briefly be summarised as follows: it possesses little or no action, on most bacteria, when in the dry state. When specimens are actually wet, they will be destroyed, except in certain cases of resistant forms, as the bacillus tuberculosis, and certain other organisms when in their resting or sporulating stage. This means that sulphur is of little value in disinfecting the apartments of consumptives, or those in which the patient suffered from a disease, the organisms producing which was known to be capable of spore formation.

To produce germicidal results the walls, floor and articles in the room should be sprayed with water, and from three to six pounds of sulphur burned to each thousand cubic feet of air space. By this procedure many articles are destroyed for future use, yet it is the only positive means of accomplishing disinfection with sulphur.

Since the introduction of formaldehyde gas, many methods have been proposed for its application and most of them include the use of complicated and expensive instruments. In the use of sulphur, the universal household method of generating the gas is by the burning of candles devised for this purpose. Until very recently, the methods suggested for applying formaldehyde have been as follows:

Fig. 1.—Formaldehyde Autoclave.

1. The use of an apparatus known as an autoclave, the gas being generated under pressure from solutions of formaldehyde incorporated with various proportions of calcium chloride. (Fig. 1.)

2. By the use of the formaldehyde gas regenerator in which formaldehyde passes from a tank into a shallow chamber, is decomposed by heat, into paraformaldehyde, the heat being sufficient to cause regeneration of the gas. (Fig. 2.)

3. By the oxidation of wood alcohol, using lamps especially constructed for the purpose. (Fig. 3.)

4. By heating a substance known as paraformaldehyde, or paraform, in an apparatus originally designed by Schering. (Figs. 4 and 5.)

5. By the vaporisation of formaldehyde containing a small percentage of borax or mixtures of formaldehyde and glycerine, in an apparatus as designed by Novy. (Fig. 6.)

6. By the use of sheets, suspended in the apartments, on which varying amounts of formaldehyde has been sprayed.

7. The use of an atomiser having a forced bulb attachment, solutions of formaldehyde being employed.

Whereas many of these methods are efficient in their germicidal effects, none of them, in the judgment of the writer, meet the requirements of simplicity in a sufficient degree to render them practical for household use and still possess the necessary dependence to insure absolute safety in the use of articles after the existence of a contagious or infectious malady.

The following series of experiments, "A" and "B," tend to demonstrate two important factors regarding the use of formaldehyde: (1) The slight penetrating properties of the gas with certain substances; (2) the unreliability of the most common method which has been employed in many series of tests to determine whether the gas has the power of surface disinfection.

Fig. 2.—Sanitary Construction Co.'s Formaldehyde Regenerator.

TEST-SERIES "A."

A fluid culture of the bacillus prodigiosus was mixed with pulverised sugar and allowed to dry. The mixture was again pulverised and dusted on the surface of the materials mentioned in such a manner that gentle shaking would easily cause its removal without actually touching the surfaces.

After being exposed for twelve hours in a room containing 2,100 cubic feet, formaldehyde having been introduced by the autoclave method, the results were as follows:

(*a*) Dusted on plaster having smooth finish, prodigiosus destroyed.

(*b*) Dusted on plaster having rough finish, prodigiosus destroyed.

(*c*) Dusted on ordinary wall-paper, prodigiosus destroyed.

(*d*) Dusted on painted wood surface, prodigiosus destroyed.

This series of tests would appear to establish, without question of doubt, the efficiency of the gas as a mere surface disinfectant. The known infected materials occupied the same position as do the walls of a dust-laden apartment.

All "control cultures" grew.

TEST SERIES "B."

The surfaces of the plaster having the smooth finish, plaster having rough finish, wall-paper and painted wood which were used in the tests of series "A" were slightly touched with sterilised platinum wires and sterilised cotton swabs, and inoculations made into nutrient gelatine, nutrient agar and bouillon, resulting in bacterial growths in all media, with the exception of those inoculated from the paint. The growths present were not the bacillus prodigiosus. This test appears to demonstrate the extremely slight penetrating properties of formaldehyde with the materials mentioned. Also the unreliability of this test method, due to the difficulty of removing portions of the surface dust without, at the same time, removing a portion of the plaster or other material under observation. All test objects were kept under glass covers on completion of the exposures to formaldehyde, thereby preventing possible contamination from outside sources.

Fig. 3.—Kuhn Formaldehyde Lamp.

Some reports published in regard to the value of certain instruments betray such a bias in favor of a particular apparatus, as to suggest that their authors, in their ambition to write up this method, lost sight of the fact that it is the practical germicidal effect which is desired, and any procedure that embraces, first of all, simplicity, and yet retains its effectiveness, is the one that will prove most useful in household application.

The autoclave method is efficient in its results, but is not simple of application. It is necessary to have a large machine of expensive construction that cannot be satisfactorily employed by an inexperienced person. This apparatus consists of a heavy silver-lined copper cylinder into which a mixture containing formaldehyde with some neutral salt—usually calcium chloride—is placed. The funnel-like valve, (Fig. 1— B) by means of which the mixture (composed of formaldehyde, 40 per cent., 1,000 parts; calcium chloride, 200 parts; water, 400 parts) has been introduced, is closed and a Swedish lamp (Fig. 1—C) applied to the bottom. By an automatic device (Fig. 1—E) when sufficient pressure is generated—usually that of three atmospheres —(temperature, 135° C) the gas is liberated through a small rubber tube, which can be conveniently introduced through a key-hole or some other small aperture, into the room to be disinfected. About seventy minutes is required to volatilise one liter of the fluid.

Fig. 4—Schering's Disinfectant Lamp.

The formaldehyde regenerator has the disadvantage of becoming readily disordered, and considerable annoyance is experienced by the plugging of the discharge tube with solid paraformaldehyde. This method likewise requires a special apparatus which, in the writer's experience could not at all times be depended upon for use. The machine is difficult for an inexperienced person to operate, but, when satisfactorily employed, is efficient in its results.

The oxidation of wood alcohol by lamps designed for this purpose has not proven successful in household application, for the reason that insufficient amounts of gas are generated to supply the necessary concentration.

A lamp known as the Kuhn formaldehyde generator, according to an opinion expressed by Dr. Geddings,[2] of the U. S. Marine Hospital Service, is more effective in its results than any other methyl alcohol lamp. This factor, if so, is probably due to the size and construction of the instrument which allows of more rapid oxidation of a much larger quantity of wood alcohol. The alcohol is placed in a receptacle at the bottom, and when lighted, its vapor passes between two cones of platinised asbestos, one of which is so arranged as to act as a deflector, thus preventing extreme heat being thrown directly upon the surface of the vessel containing the wood alcohol. Any of the alcoholic vapor which escapes the platinised cones passes through five disks or layers of platinised wire. In this way, it is thought, the alcohol is brought into more certain contact with platinised surfaces, which is absolutely necessary in the

Fig. 5—Schering's Disinfector.

conversion of the alcohol into formaldehyde gas. Lack of personal experience with this device does not permit of the confirmation of the results said to have been attained in its use in the Marine Hospital Service. If the generator accomplishes what is claimed for it, it is the only methyl alcohol lamp on the market which is at all serviceable in household disinfection. Allowing that this method is efficient, it, like the others, requires an expensive apparatus for carrying on the operation.

Fig. 6.—This Machine can be used as an Autoclave and also for Vaporisation of Mixtures.

The method suggested by Schering is efficient in results, but personal experience demonstrates that a much greater number of pastiles must be volatilised than is suggested by the dealers. The method, likewise, requires a special apparatus for carrying on the operation, but the device is inexpensive compared with the former appliances mentioned. The mode of using the apparatus is simple.

The disinfector (Fig. 5) or the disinfecting lamp (Fig. 4) is placed upon a sheet of iron on the floor of the room to be disinfected. In the disinfector about 200 pastiles can be evaporated at a time. For the production of greater quantities of formaldehyde vapor, several outfits must be employed. The pastiles are placed in the cup-like attachment in the top and heat is supplied by the use of an ordinary spirit lamp.

The disinfector is supplied with a small boiler-like arrangement which answers the purpose of furnishing moisture with the gas. Seventy-five pastiles are advertised as being sufficient for the surface disinfection of each 1,000 cubic feet of space. Personal experience has demonstrated that not less than 200 pastiles should be relied upon for this result. In apartments containing more than 5,000 cubic feet, a hospital ward, and the like, it has not been possible for the writer to insure surface disinfection by the use of 200 pastiles to every 1,000 cubic feet of air space.

The vaporisation of mixtures of formaldehyde with either borax or glycerine has, in the use of the glycerine, the disagreeable feature of covering all articles in the room with a sticky substance which, in many instances, causes destruction of the materials. When used with borax, it is said not to possess this feature and to be equally efficient in its germicidal results. The method, likewise, requires the use of a special apparatus.

Fig. 7.

Personal experience with the use of sheets, sprayed with 40 per cent. solutions of formaldehyde, using 180 c.c. to 1,000 cubic feet of air space, has not given uniformly constant results. This method, however, has been adopted by the Chicago Board of Health, and reports from that department appear satisfactory.

The use of the spray, applying solutions of formaldehyde directly upon the articles to be disinfected, is a most difficult operation, on account of the extremely irritating properties of the gas, affecting, most noticeably, the eye and air-passages of the operator. This method has been adopted by the Pullman Palace Car Company in the disinfection of its cars.

It is possible for sulphur fumigation to be efficiently carried out by the most inexperienced operator and sulphurous acid gas is unquestionably a valuable bactericide, but the ordinary household application, as it is practised in Buffalo, is of restricted value, in that the articles, in most instances, are not previously sprayed with moisture. Sulphur dioxide has accomplished much in the past, and undoubtedly will continue to do so in the future, but experience has demonstrated formaldehyde to be a far less destructive and an equally potent and reliable germicide.

There has recently been placed upon the market a candle (Fig. 7) consisting of paraformaldehyde which can be applied in the same manner as are those composed of sulphur. This candle consists of paraformaldehyde incorporated with a small proportion of paraffine and pressed in cylindrical form, the cylinders being of two different sizes. To utilise the candle, it is supplied in a tin container or burner to which a limited amount of oxygen has access during operation so as to support combustion only at the bottom of the candle, and by burning in this manner the heat produced causes the solid paraformaldehyde to revert to the gaseous formaldehyde.

Paraform burns freely and the flame would extend over the entire surface if some container limiting the supply of oxygen during combustion was not employed. If the entire surface of the candle was allowed to burn, the gas would be converted into carbon dioxide and water by the flame and rendered inert for disinfecting purposes. The smaller size candle contains, approximately, 350 grains of paraformaldehyde, and according to tests (series C. and D.) carried on in the Bureau of Bacteriology, Department of Health, Buffalo, N.Y., this candle when properly ignited, generates sufficient gas for the surface disinfection of a room not exceeding 300 cubic feet capacity, and by increasing the number of candles in the proportion of one to every additional 300 cubic feet of air space, this method can be relied upon for the surface disinfection after certain diseases (the limitations of which are explained later) in rooms not exceeding 3,000 cubic feet capacity. With this, as with all other methods, if the room capacity greatly exceeded this amount, even though an additional number of candles in the same ratio were applied, it was impossible to obtain by sufficient rapid diffusion the necessary concentration of the gas to insure constant results. This may, in a measure, explain the reason for failures in the use of formaldehyde gas to act as a surface disinfectant in many instances reported.

As regards the tests conducted at the Army Barracks, Fort Meyer, Va.,[3] those made by the writer in series "B" account for the results obtained. In this instance, the tests conducted were by touching the walls with platinum wires and subsequent culture of material obtained. The apartment in which many of the experiments were conducted at the Department of Health, Buffalo, was such as would represent a living room of a capacity of about 2,100 cubic feet of air space and contained three large windows with moderately tight casings, and no additional precautions were taken to prevent the escape of the gas. In using the larger candle which contains, approximately, 700 grains of paraform, one candle was found to be sufficient for 500 cubic feet of air space and efficient up to a capacity not exceeding 3,000 cubic feet.

To insure the best results, in the use of this method of room disinfection, the following precautions should be exercised: (1) The room made as nearly air-tight as possible; (2) The use of one small candle to each 300 cubic feet of air space, and not depending upon this means of disinfection in apartments containing more than 3,000

cubic feet; (3) that the surfaces of the articles to be disinfected be so arranged as to allow free exposure to the gas; (4) that the room remain closed from six to twelve hours; (5) that a cleansing process supplement the procedure.

Certain facts are to be particularly remembered in the application of this gas in household disinfection. While diffusing with great readiness, it possesses but slight penetrating properties. This is easily shown by the facility with which certain bacteria were destroyed in test series C and D when distributed through porous fabrics, and yet retain their ability to grow in cultures when spread upon surfaces like glass, metal and glazed cardboard.

These facts demonstrate that to thoroughly disinfect a room and its contents, we should not depend solely upon formaldehyde, but that, in rooms having glazed surfaces, china, marble, metal, and the like, subsequent washing with solutions possessing recognised germicidal properties, like 5 per cent. solution of formalin, 5 per cent. carbolic acid, etc., should be employed. Thus, it is shown that while in formaldehyde gas we now have an efficient and harmless disinfectant for such objects as laces, plush, velvet, curtains, table-covers, hangings, and the like, for which heretofore no satisfactory household method of disinfection had been devised, and while in many cases its action upon all objects may be efficient and satisfactory, and its action upon metals, picture frames, and so forth, harmless and non-corrosive, in cases of serious infection, where it is possible that infectious matter, such as blood, saliva, sputum, feces, pus or urine may be dried upon wood floors, chamber vessels, basins, washstands, glasses, metal bedsteads and other hard surfaces, its action should be supplemented by a cleansing and disinfecting solution possessing solvent and penetrating powers.

TEST SERIES "C."

Cultures of organisms named dried on material given and exposed six hours:

| | Cholera Culture. | Typhoid Culture. | Diphtheria Culture. | Diphtheria Membr'e. | B. Icteroids Culture. | B. Pestis Culture. | Tubercular Sputum. |
|---|---|---|---|---|---|---|---|
| Metal plates . . . . | o | + | + | + | ? | o | + |
| Glass . . . . . . . . | o | + | + | + | ? | o | + |
| Filter paper . | o | o | o | | o | o | o |
| Glazed cardboard . | o | + | o | | o | o | + |
| Cheese cloth . . . . | o | o | o | | o | o | o |
| Cotton batting . . . | o | o | o | | o | o | + |
| Flannel . . . . | o | o | o | | o | o | o |
| Cotton Cloth . . | o | c | o | | o | o | o |

TEST SERIES "D."

Materials impregnated with fluid cultures, and moist during time of exposure:

| | Cholera Culture. | Typhoid Culture. | Diphtheria Culture. | B. Icteroids Culture. | B. Pestis Culture. | Tubercular Sputum. |
|---|---|---|---|---|---|---|
| Metal Plates . . . . | o | o | + | o | o | + |
| Glass . . . . . . . . . | o | o | + | o | o | + |
| Filter Paper . . . . . | o | o | o | o | o | |
| Glazed cardboard . . | o | o | o | o | o | |
| Cheese cloth . . . . | o | o | o | o | o | |
| Cotton batting . . . | o | o | o | o | o | |
| Flannel . . . . . . . | o | o | o | o | o | |
| Wool Yarn . . . . . | o | o | o | o | o | |

In series "D" the infected test objects were placed in a Novy anaërobic jar. The formaldehyde candles were used in a cupboard in proportions of 1½ grains to each cubic foot of space. The gas in the cupboard was drawn through the jar and the jar closed.

By this procedure, the test objects remained moist the majority of the time during exposure, which demonstrates that moisture facilitates disinfection.

The application of formaldehyde to household disinfection can be briefly summarised as follows: (1) it is the most satisfactory of the gaseous disinfectants; (2) its penetrating powers are extremely slight; (3) a certain degree of moisture facilitates its action; (4) it should always be supplemented by a cleansing process.

The writer desires to express his appreciation to Dr. Eugene Wasdin, of the Marine Hospital Service, for furnishing the cultures of the bacillus icteroides and bacillus pestis, and to the various manufacturers for the cuts so generously loaned in publishing this article.

REFERENCES.

1. Personal conversation with Dr. Wasdin.
2. Public Health Reports—Marine Hospital Service (Vol. XV., No. 42.).
3. Personal conversation with Dr. Vertner Kennerson.

143 ELMWOOD AVENUE.

---

OSTEOPATHS in Milwaukee have been arrested on warrants sworn out by the assistant district attorney. They are charged with unlawful use of the title of "Doctor" and of practising medicine without a license from the State Board.—*Medical Age.*

## Figures and Tables

**Fig. I. f1:**
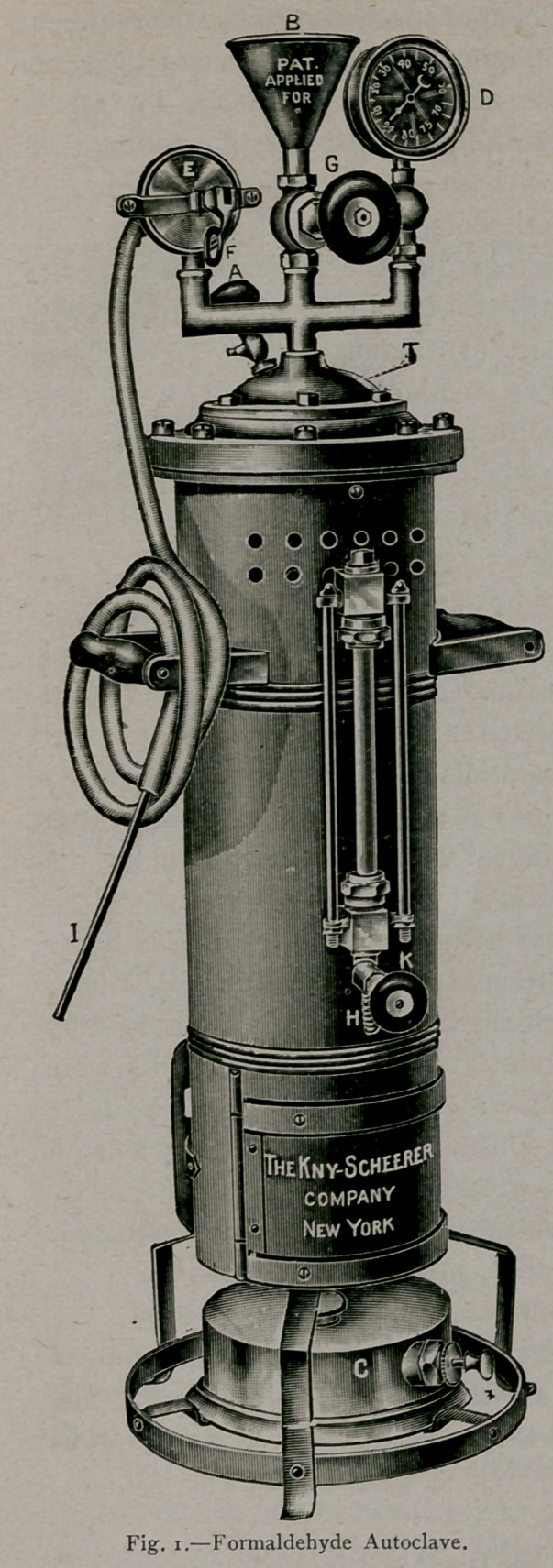


**Fig. 2. f2:**
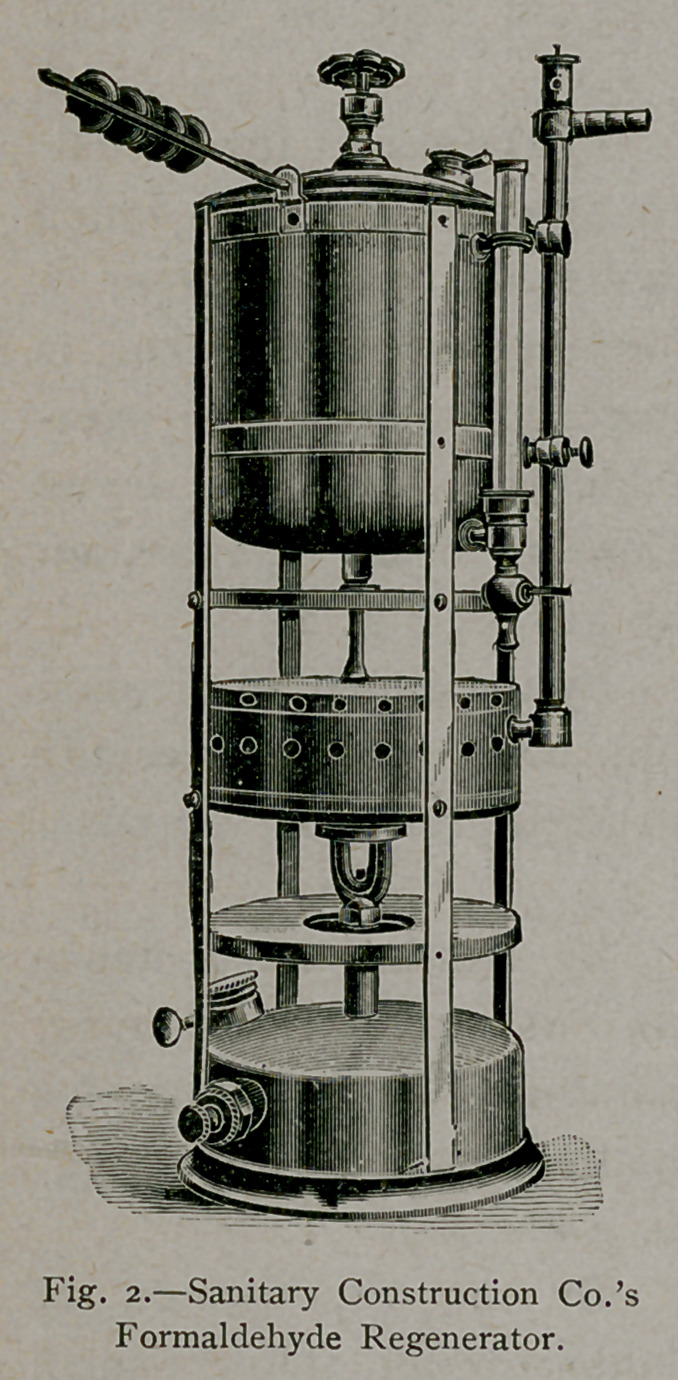


**Fig. 3. f3:**
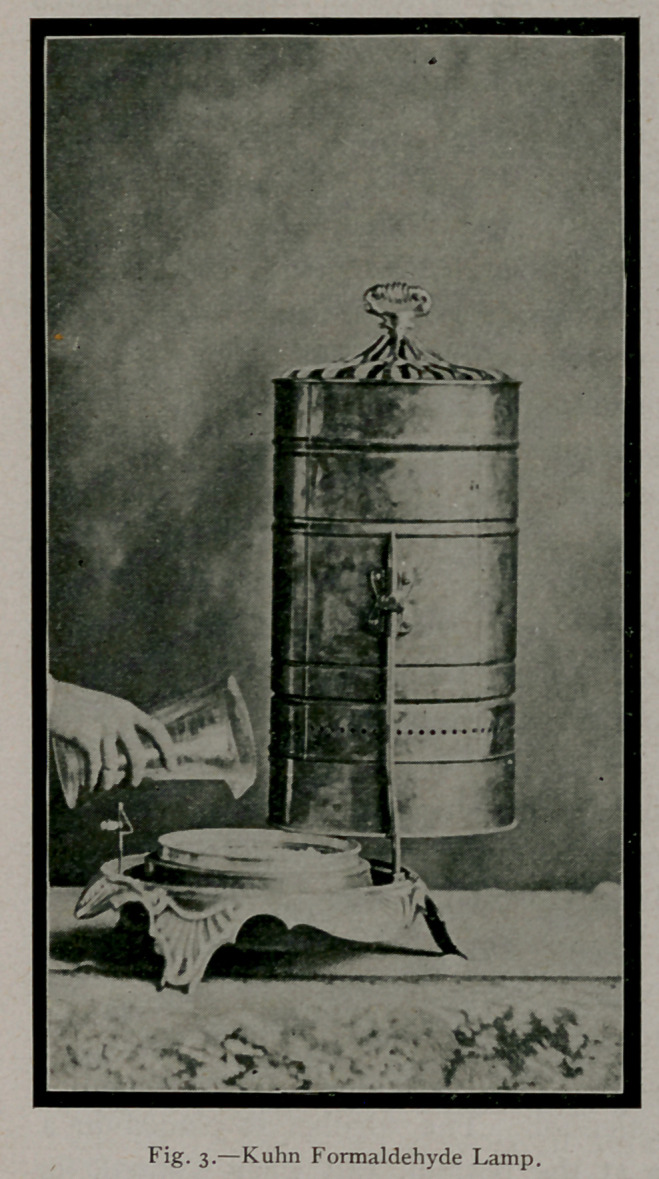


**Fig. 4. f4:**
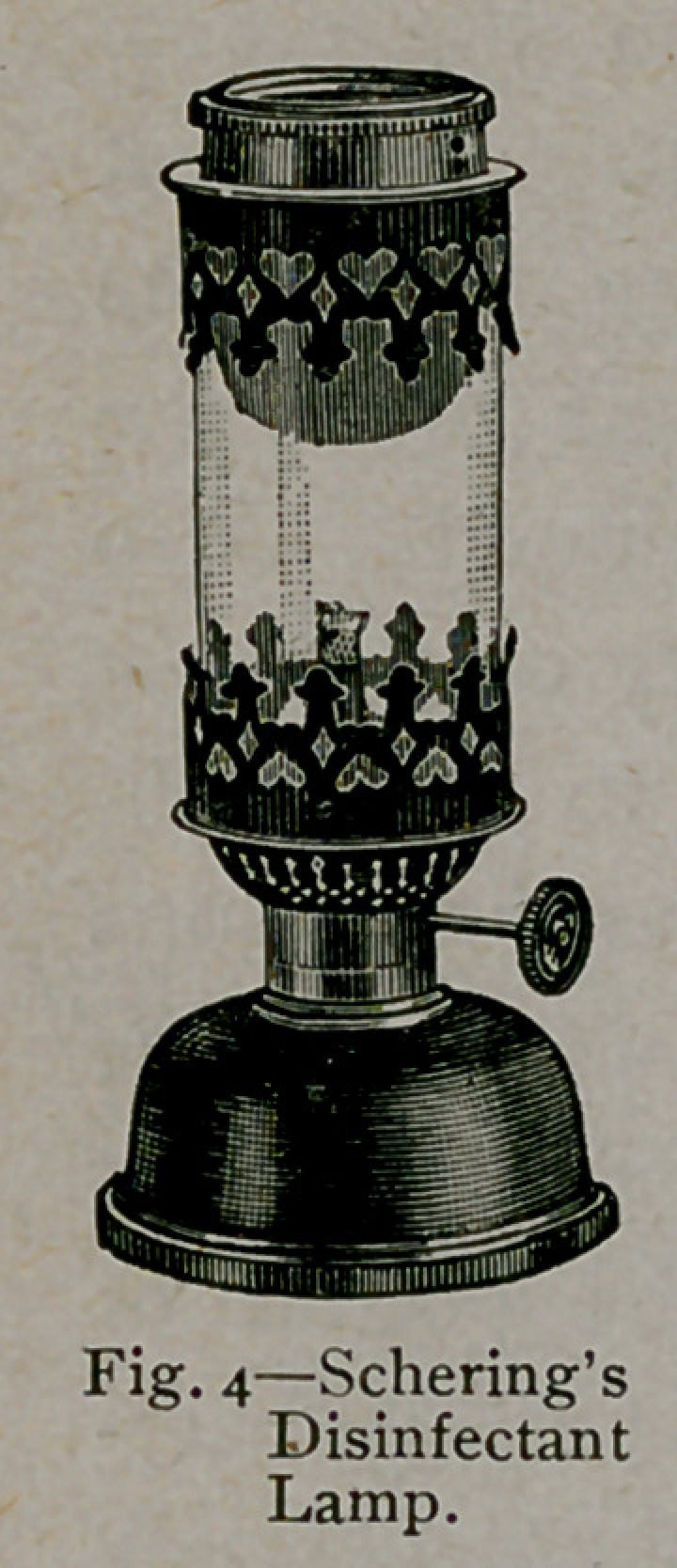


**Fig. 5. f5:**
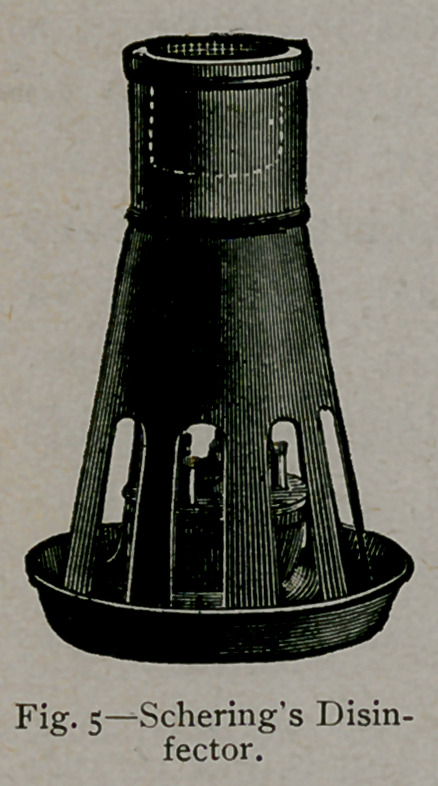


**Fig. 6. f6:**
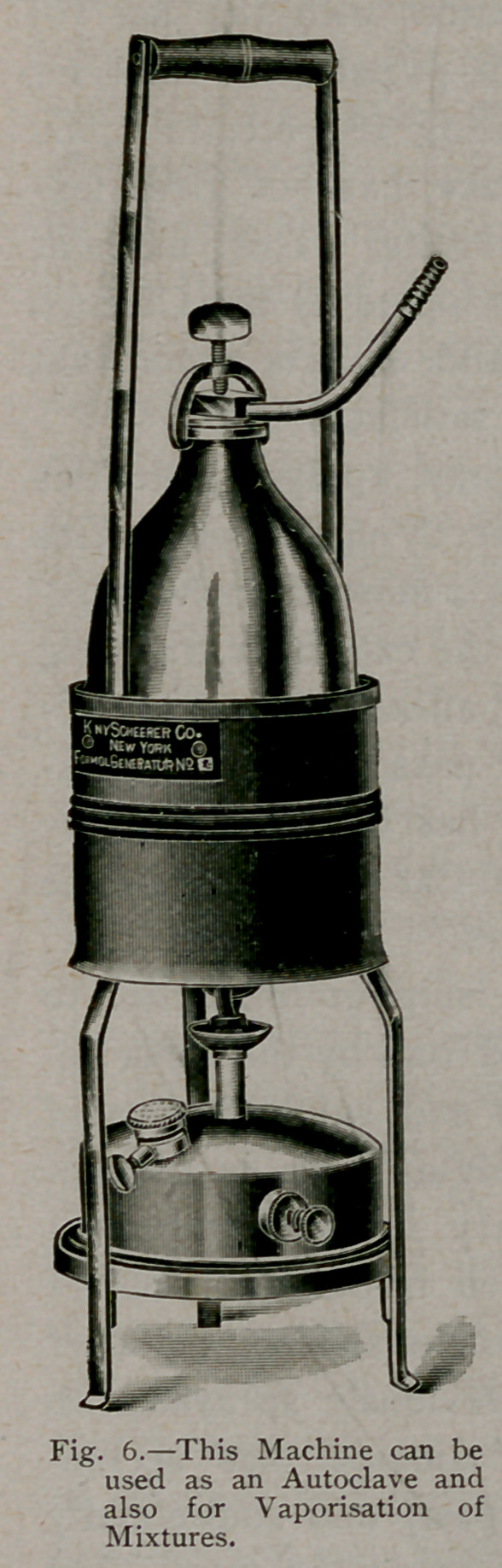


**Fig. 7. f7:**